# Dietary Fruit By-Products Improve the Physiological Status of Nile Tilapias *(Oreochromis niloticus)* and the Quality of Their Meat

**DOI:** 10.3390/antiox12081607

**Published:** 2023-08-14

**Authors:** Andrey P. Chotolli, Victor E. da Fonseca, Rubén Bermejo-Poza, Isabella G. Ferraz, Letícia C. C. de Souza, Mariana L. Brasil, Ronnie F. Santana, Isadora M. M. Games, Murilo C. Ferraz, Gabrielly Theophilo, Pedro H. L. Salmaso, André L. S. Balbino, Filipe D. R. dos Santos, Elisa H. G. Ponsano

**Affiliations:** 1 Department of Animal Health and Production, Faculty of Veterinary Medicine, São Paulo State University Unesp, 793 Clóvis Pestana, Araçatuba 16050-680, Brazil; andrey.chotolli@unesp.br (A.P.C.); v.fonseca@unesp.br (V.E.d.F.); isabella.galli@unesp.br (I.G.F.); leticia.caldas@unesp.br (L.C.C.d.S.); m.brasil@unesp.br (M.L.B.); ronnie.santana@unesp.br (R.F.S.); isadora.games@unesp.br (I.M.M.G.); murilo.ferraz@unesp.br (M.C.F.); gabrielly.theophilo@unesp.br (G.T.); pedro.salmaso@unesp.br (P.H.L.S.); andre.balbino@unesp.br (A.L.S.B.); filipe.dias@unesp.br (F.D.R.d.S.); 2 Department of Animal Production, Faculty of Veterinary, Complutense University of Madrid, Puerta de Hierro s/n, 28040 Madrid, Spain; rbermejo@ucm.es

**Keywords:** acerola, apple, carotenoids, grape, lipid peroxidation, phenolic compounds

## Abstract

By-products from fruit industrialization retain nutritional and functional components; thus, they may find use in animal feeding. This study aimed to assess the effects of dietary fruit industrial by-products on the tilapias blood biochemical and oxidative parameters and on the composition and lipid peroxidation of their fillets. Four diets were supplied to the tilapias: a C-control diet, with no fruit meal, and three diets containing 5% of either acerola (ACM), apple (APM) or grape (GRM) meal. The phenolic compounds and the carotenoids in the meals and their antioxidant capacities were measured. Fish were weighed and measured for the calculation of the growth performance data, their blood was analyzed for health and oxidative status biomarkers and their fillets were analyzed for proximal composition and lipid peroxidation. Grape meal had the highest concentration of phenolics and carotenoids and the highest antioxidant activity, followed by acerola and apple meals. The productive performance was similar among the treatments. The fruit by-product diets either maintained or improved the biochemical biomarkers of health and improved the oxidative status of the fish. The fruit by-product diets increased the concentration of lipids in the fillets and slowed down the onset of the lipid peroxidation during frozen storage.

## 1. Introduction

Intensive fish farming has been growing continuously, requiring alternatives to increase production and productivity. In view of the expected population increase for the coming years [1], the search for sources of animal diet ingredients that do not compete with human food and contribute to increase the food security, proposed among the Sustainable Development Goals established by the United Nations for 2030 [2], has been gaining strength in productive, academic and industrial circles.

Nile Tilapia (*Oreochromis niloticus*) is the current highlight in world aquaculture. In 2022, Brazil contributed 550,060 tons of the 6.5 million tons of tilapia produced in the world, ranking fourth in the world as a producer of the species [3]. Tilapia meat has great consumer acceptance due to its mild flavor, light color, absence of bones and ease of preparation [4]. Brazil also ranks third in the world for fruit production, accounting for approximately 41 million tons in 2022 [5]. A significant part of the fruit is industrialized, generating by-products that retain nutritional and functional properties and deserve sustainable usage, for example, in aquafeed production [6].

The use of fruit by-products to replace part of the main components of fish diets can improve food security, reduce the environmental impact and the costs of animal products [7], besides improving the animal health due to the presence of bioactive compounds with antioxidant, anti-inflammatory, antimicrobial, hypoglycemic and immunostimulant properties, such as phenolic compounds and carotenoids [8,9]. This use perfectly applies to the principles of a circular economy, which include reducing the amount of waste generated in the food systems, the re-use of food, the utilization of by-products and food waste, nutrient recycling and more diverse and efficient food patterns [10].

Phenolic compounds are single molecules or polymers made up of an aromatic ring linked to one or more hydroxyl groups, originated from the secondary metabolism of plants [11,12]. Many phenolic compounds have antioxidant activity due to their ability to capture and stabilize free radicals involved in oxidative processes [13].

Carotenoids are natural pigments synthesized by photosynthetic organisms such as plants, algae and cyanobacteria, as well as by some fungi and bacteria [14]. Most animals cannot biosynthesize carotenoids, although they can absorb them from the diet and incorporate them in different tissues [15]. Chemically, they are polymers of isoprene, either made up solely of carbon and hydrogen or containing oxygenated substituents [16]. Carotenoids show robust antioxidant activity, scavenging and inactivating free radicals and preventing or reducing damage caused by these compounds in cells [17,18]; thus, they are considered important micronutrients that should be included in animal diets [19].

Free radicals are chemical compounds with unpaired electrons, unstable and highly reactive, originating from the endogenous aerobic metabolism or from exogenous factors [20]. If not combated, they initiate the oxidative process, damaging lipids, proteins and DNA and affect the cell structure and function [21,22], besides producing toxic compounds that may cause blood injury and membrane lesions [23].

Diet, temperature, stocking density and water quality are stressing conditions that may lead to oxidative stress in farm-raised fish [24], causing physiological and histopathological damage, immune deficiencies and decreased meat quality due to oxidative reactions [25,26]. These reactions take place in proteins and lipids, destroying nutrients, altering taste, odor and texture and even generating toxic compounds, which result in food rejection by the consumer and economic losses [27,28].

To minimize such occurrences for the animals and their edible products, natural or synthetic antioxidants may be added to their feeds. The dietary antioxidants incorporate into the cell membranes, neutralizing the active forms of reactive oxygen species involved in the initiation and propagation steps of lipid oxidation, thus increasing animal growth, improving resistance to stress and diseases and maintaining the meat quality [29,30]. Although the use of synthetic antioxidants in foods and feeds is approved by health regulatory agencies and is under constant evaluation, there is a concern among consumers regarding their effects on health [31]. Thus, the use of natural antioxidants in animal diets meets the growing interest of consumers for healthier foods [32]. 

In the case of fish farming, the use of natural feed additives is encouraged by the World Health Organization (WHO) and the Food and Agriculture Organization of the United Nations (FAO) as a way of boosting immune response and decreasing the occurrence of diseases related to the intensive production, thus avoiding economic losses [33]. The roles of supplementing aquafeed with apple, banana, citrus and grape by-products were recently reviewed and, in general, improvements in health and growth performance, immunity, antibacterial activity and total antioxidant status of different aquatic animals were described [34]. By-products from papaya, passion fruit and pineapple were also studied as feed additives in aquaculture resulting in positive results for fish and shrimp farming and reinforcing issues on waste recycling, waste reduction and competition with human food [35].

The effects of using fruit by-products as additives in fish feed require the evaluation of health indicatives, and the monitoring of the blood biochemical characteristics may help in the detection of any physiological or pathological change associated with their consumption [36,37]. Moreover, some fruit by-products may contain antinutritional factors that negatively affect nutrients bioavailability in the digestive system, compromising fish health and performance. Thus, the inclusion of fruit by-products in fish diets deserves investigation on several aspects related to animal growth, health and welfare and to the quality of the edible product.

This study aimed to quantify the bioactive compounds and their antioxidant capacity in by-products of apple, acerola and grape industrialization and assess the effects of their dietary intake on the blood biochemical and oxidative parameters of Nile Tilapias and on the composition and the lipid peroxidation of their fillets. 

## 2. Materials and Methods

### 2.1. Experimental Design, Diets and Management

The trial was carried out in a completely randomized design, with four treatments and six replicates. The treatments included a control (basal) diet (C) and three experimental diets containing either acerola (*Malpighia emarginata*, ACM), apple (*Malus domestica*, APM) or grape (*Vitis vinifera*, GRM) meal at 5%. The diets were nutritionally equivalent and were formulated to reach the requirements for tilapias according to the Brazilian Tables for the Nutrition of Tilapias [38] using the software NUTRIMAX version 13.10 [39] (Table 1). The fruit meals were obtained from Brazilian juice industries and had the following mean composition (moisture, proteins, lipids, fibers, ashes and carbohydrates, respectively) in % dry matter: acerola meal: 9.46, 7.38, 1.31, 61.83, 1.92, 18.1; apple meal: 2.84, 5.71, 5.62, 60.53, 2.26, 23.04 and grape meal: 5.46, 12.25, 11.11, 69.30, 1.79, 0.09.

Commercial tilapias were distributed in 24 plastic 1000 L tanks (12 fish/tank; initial body weight per tank 4174 ± 361.92 g) interconnected in a closed water recirculation system with forced aeration distributed by porous stones. The water quality was controlled by siphoning the bottom of the tanks, partial water exchange (30%) and monitoring of temperature (infrared thermometer), pH, nitrite, ammonia and dissolved oxygen with Labcon Test kits (Alcon, Camboriú, Brazil) twice a week (mean values: temperature 23.89 ± 0.71 °C; pH 7.17 ± 0.04; nitrite 1.68 ± 0.8 ppm; ammonia 2.46 ± 0.29 ppm and dissolved oxygen 9.78 ± 0.58 ppm). 

All the fish were anesthetized with eugenol at 40 mg/L, weighed and measured at the beginning and at the end of the experiment. The experimental diets were supplied for 45 days at 6% of body weight, divided in two and given twice a day (8 a.m. and 5 p.m.). The natural photoperiod was adopted during the trial time. In the case of mortality, the tank body weight and the feed supply were corrected. The procedures for rearing, stunning and slaughtering were approved by UNESP Ethics Committee on the Use of Animals (CEUA 0143-2021).

### 2.2. Determination of Bioactive Compounds and Antioxidant Activity in the Fruit Meals

The phenolic compounds were first extracted with 50% methanol in the dark for 60 min. After centrifugation (2863× *g*/30 min), the supernatant was filtered and reserved. The residue was re-extracted with 70% acetone in the dark for 60 min and centrifuged with the same conditions. The ketone supernatant was filtered and added to the alcoholic extract. The determination of total phenolic compounds was accomplished by the Folin–Ciocateu method, using a standard curve of gallic acid [40]. 

Before the extraction of the carotenoids, 0.1 g of each fruit meal was added of dimethyl sulfoxide and incubated in an ultrasound bath at 55 °C for 30 min. For the extraction, cycles of solvent addition (acetone)/vortex agitation (1 min)/centrifugation (3400× *g*/10 min) were repeated until the residues became colorless. Water and ether were added to the pooled supernatants until partition. The ethereal phase containing the carotenoids was removed and let to dry. The solid carotenoids were resuspended in 2 mL acetone and their absorbance was read at 475 nm against acetone as a blank. Total carotenoids were determined in mg/0.1 g from the expression (Abs_475_ × 2 × 10)/2500, where 2500 is the coefficient of extinction for total carotenoids (adapted from [23]).

The oxidant activity was determined in the extracts by 2,2′ azino-bis (3-ethylbenzothiazoline-6-sulfonic acid) (ABTS) and 2,2-diphenyl-1-picrylhydrazyl (DPPH) methods [41,42]. The results were expressed in µM Trolox/g and in efficient concentration (EC50, mg/L), respectively, for ABTS and DPPH.

### 2.3. Stunning, Slaughtering and Sampling Procedures

Four fish from each repetition of each treatment (24 fish/treatment) were randomly selected, anesthetized (eugenol 40 mg/L) for the collection of blood from the tail veins and slaughtered by cross spinal section. Heads, tails and viscera were removed and discarded; the livers were removed and weighed, and the fillets were removed for the chemical analyses.

### 2.4. Determination of Growth Performance

Feed consumption was calculated based on the amount of feed supplied. Weight gain (WG), hepatosomatic index (HI) and feed efficiency index (FE) were calculated as:

WG = final weight (g) − initial weight (g)(1)

HI = (liver weight (g)/total weight (g)) × 100(2)

FE = average daily weight gain (g)/average daily feed intake (g)(3)

Body condition factor (BC) was calculated from the model W = aL^b^, where W = weight (g); L = length (cm); a = coefficient determined by fitting the model to observations of length at weight and b = allometric growth parameter [43].

### 2.5. Determination of the Blood Biochemical and Oxidative Parameters

Blood serum was analyzed for urea (UV enzymatic method); creatinine (kinetic method with alkaline picrate); albumin (bromocresol green method); total protein (TP: biuret method); cholesterol (oxidase/peroxidase enzymatic method); uric acid (UA: uricase/peroxidase enzymatic method); alkaline phosphatase (AP: diethanolamine method); alanine aminotransferase (ALT: conversion of amine groups to pyruvate method) and aspartate aminotransferase (AST: conversion of amine groups to oxalacetate). Globulin was determined by difference (total protein–albumin). The total antioxidant status (TAS) was measured by the colorimetric method of ABTS cation inhibition, and the total oxidant status (TOS) was measured by the colorimetric method of Xylenol Orange [44,45]. The analyses were performed at 37 °C using commercial reagents (BioSystems, Barcelona, Spain) and an automated biochemical analyzer (BS-200 Chemistry Analyzer, Mindray Bio-Medical Electronics Co., Shenzhen, China) calibrated with commercial calibrators and controls (Bio Systems, Barcelona, Spain). Lipid peroxidation was measured by the thiobarbituric acid reactive substances (TBARSs) method with commercial reagents (ZeptoMetrix Corporation, Buffalo, NY, USA) and automatic microplate reader (Readwell Touch, Robonik PVT LTD, Thane, India) [46].

### 2.6. Determination of the Fillets Proximate Composition and Lipid Peroxidation 

The meat was ground in a food processor for the next analyses. Moisture was determined at 105 °C until constant weight; ashes were determined at 550 °C; proteins were determined by the micro-Kjeldhal method (protein conversion factor 6.25) [47]. Lipids were determined with the cold extraction technique [48] and the carbohydrate concentration was obtained by difference. The lipid peroxidation of the fillets frozen at −20 °C was determined by the quantification of the thiobarbituric acid reactive substances (TBARSs) at days 10, 30 and 60 after slaughter [49].

### 2.7. Statistical Analysis

The statistical analyses were performed with the Jamovi Statistical Program version 2.2.5 [50]. A prior analysis of the normality and homogeneity of variance of all data was performed using Shapiro–Wilks and Bartlett tests, respectively. One-way analysis of variance (ANOVA) was conducted using the diets as the fixed effect. Differences between means were analyzed by Tukey’s test (*p* < 0.05). 

## 3. Results

### 3.1. Bioactive Compounds and Antioxidant Capacity in the Fruit By-Products

Grape meal had the highest concentrations of total phenolic compounds and carotenoids and the best antioxidant capacity by both methods (ABTS and DPPH) (Table 2). Since EC50 means the amount of the fruit meal necessary to reduce the concentration of the DPPH radical by 50%, the lower this value, the higher the antioxidant activity [51].

### 3.2. Growth Performance

With the exception of the highest weight gain detected in the tilapias fed acerola meal, the dietary by-products did not exert influence on the growth performance of the animals (Table 3).

### 3.3. Blood Biochemical and Oxidative Parameters of the Tilapias

Blood biochemical and oxidative parameters are shown in Table 4. Total protein, albumin, alanine aminotransferase, aspartate aminotransferase, cholesterol and triglycerides were not altered by the diets. The control diet provided the highest TBARSs and total oxidant status well as the lowest total antioxidant status in the fish blood, evidencing the antioxidant property of the bioactive compounds in the fruit by-products.

### 3.4. Proximate Composition and Lipid Peroxidation in the Tilapias’ Fillets

**Figure 1:**
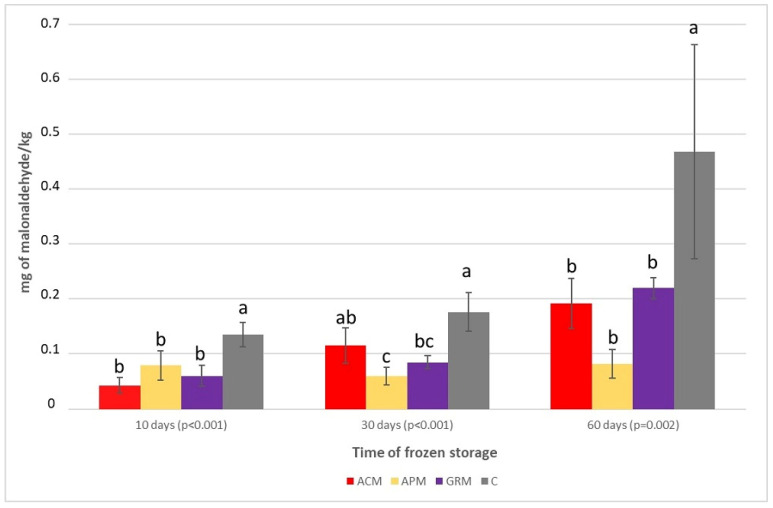
Lipid peroxidation (mg malonaldehyde/kg) in the fillets of tilapias fed the experimental diets at different times of storage at −20 °C (means ± standard deviation). ACM = diet with 5% acerola meal; APM = diet with 5% apple meal; GRM = diet with 5% grape meal; C = control diet; bars followed by different letters differ significantly by Tukey’s test (*p* < 0.05).

The lipids concentration increased in the fillets of the tilapias fed the fruit by-products, and the protein concentration was also increased in the fillets of the tilapias fed the grape meal (Table 5). The antioxidant compounds of the diets containing the fruit by-products decreased the lipid peroxidation and reduced the TBARS concentration in the frozen tilapia fillets at all analyzed times. At 60 days of frozen storage, the TBARS concentration in the control group was more than double the concentrations found in the groups that received the diets containing the fruit by-products (Figure 1).

## 4. Discussion

### 4.1. Animal Performance

Due to the essential role nutrition plays on animal health and welfare [52], the effects of any change in the diet should be carefully investigated on the productive parameters before implementation. It is expected that the dietary bioactives improve the animal performance by combating free radicals resulting from the high metabolic rate of growing tissues, as it occurs in production animals. They can also act in the preservation of vitamins with the antioxidant activity of the diet, in the control of inflammatory processes and pathogenic intestinal microorganisms and in the modulation of the immune response, thus reflected in better performance [53]. 

Although the tilapias fed ACM had the highest weight gain, no other changes in the productive performance parameters were found to the other groups. It is reasonable to conclude that the concentration of the fruits’ meals and/or the feeding time were not enough to evidence these expected improvements. Conversely, some polyphenols may act as antinutritional factors, inhibiting digestive enzymes, increasing protein excretion, decreasing the digestibility of proteins and amino acids and exerting adverse metabolic effects that result in decreased body weight and feed efficiency [54], but none of these effects were detected for the tilapias fed the fruit by-products.

Feed efficiency allows assessing whether a feed meets the animals’ specific nutritional requirements for maintenance and production [55]. Thus, the higher the FE, the greater the efficiency in the use of the feed by the animal [56]. As FE was equivalent among the treatments, we concluded that the nutrients in the fruit by-products were efficiently used by the tilapias, with no prejudice of any possible antinutritional factor. The hepatosomatic index is another useful physiological indicator of food utilization since the liver growth reflects the animal metabolic demands. Increased HI suggests lipid deposition in the liver due to metabolic factors, such as increased gluconeogenesis or stressors [57], which were not evidenced in our results. 

Body condition factor is a good quantitative indicator of the degree of fish welfare and is, therefore, used to assess on feeding supply, stocking density, climate and other environmental conditions in fish farming [58]. Fish BCF ranging from 2.5 to 3.5 indicates the proportionality between weight and length during the growth [59]. So, the closer the BC is to 3.0, the more isometric the growth; thus, our results indicated a positive effect of the fruit by-products on the tilapias` growth, which, at the end, means economic gains.

### 4.2. Biochemical Parameters

Blood biochemical parameters are also used as biomarkers of animal health and welfare [60]. The inclusion of the fruit meals in the tilapias’ diets did not significantly affect the concentration of total blood proteins (albumin + globulin) and albumin levels, thus suggesting the health condition of the fish. Albumin makes up 35 to 50% of the blood’s total proteins and carries important roles in the muscle metabolism and the immune system [61]. Globulins comprise proteins with a role in the transport and/or immune systems and as enzymes [62]. Low concentrations of globulins in the blood, as found in the APM group, imply a low need for immune system activity due to low stress [63]. 

Alkaline phosphatase indicates hepatobiliary injury [64], and the increased values found in the fish of group C were compatible with the presence of stones detected in their gallbladder at slaughter. This finding confirmed the hepatoprotective activity of the phenolic compounds of the fruits’ meals [65], since the stones were not found in the other groups. However, the increase of alkaline phosphatase in group C was not accompanied by the increase in the concentrations of AST and ALT, which act in the regulation of hepatic function and were expected to be elevated in the case of injury to the hepatocytes [66]. The normality of AST and ALT may also indicate the good ability of the fish in metabolizing the proteins of the diets [67].

Some phenolic compounds, such as quercetin, are associated with a decreased glycemic index because they can increase the sensitivity to insulin, thus decreasing the level of serum glucose [68,69]. Although grapes are recognized as a source of quercetin [70], the grape meal used in the tilapias’ diet was not able to reduce blood glucose.

The level of cholesterol in the blood correlates with high metabolic rates of lipids, which occurs mainly under stress conditions [6]. The cholesterol levels within the reference limits in all groups indicated the maintenance of the tilapias’ homeostasis. Moreover, there is also a positive correlation between blood cholesterol and feed efficiency rate [55], which confirmed the nutritional adequacy of the experimental diets. Altered levels of triglycerides may indicate dysfunctions in the metabolism of lipids and lipoproteins [71,72,73], but it did not happen in this study. 

High concentrations of uric acid may indicate the malfunctioning of the kidneys and liver, causing hyperuricemia and failures in the animal’s metabolism [74]. The high levels of uric acid and the increased alkaline phosphatase in fish fed the control diet reinforced the liver implication, as well as the protective function of the fruits’ bioactive compounds for the fish livers [65]. 

As creatinine is excreted by the kidneys and deposited in the muscles, high concentrations of the metabolite may indicate malfunctioning of this organ [75], which was not detected in any of the treatments. Urea is an important indicator of the metabolic status of fish, as it is associated with the utilization of the dietary protein [76]. In fish, urea is excreted via the urine or the gills [77], so increased concentrations may indicate gill epitheliopathy and/or renal diseases [64]. The highest levels of urea were found in group C, thus reinforcing the beneficial effects of the bioactive compounds of the fruits’ by-products for the fish health [78].

To conclude, all the blood biochemical parameters found agreed with the reference limits for tilapias [36,79,80]. 

### 4.3. Oxidative Status

Cellular oxidative stress occurs when the oxidative forces overcome the intrinsic antioxidant systems [81]. In this process, the active oxygen forms may cause lipid peroxidation, protein carbonyl formation, muscular and DNA damage [82,83]. TBARS, TAS and TOS tests measure the amounts of products derived from the cellular oxidative stress. So, it can be said that the highest TBARS and TOS and the lowest TAS levels in the blood of the fish fed with diet C indicated the worst antioxidant activity and highlighted the antioxidant activity of the bioactive compounds of the dietary fruits’ meals. 

The phenolic compounds infiltrate hydrophobic (such as cellular lipid bilayers) and hydrophilic (such as blood serum) areas, causing a positive cell modulation [13,84], increasing the redox potential, acting as hydrogen donors and attracting oxygen molecules, thus preventing the formation of free radicals and increasing the intrinsic antioxidant properties [85]. Dietary carotenoids can deposit in different organs and tissues of animals, such as muscles, fat, skin and blood and act as antioxidants [86] preventing lipid peroxidation, reducing cellular oxidative stress and inflammatory responses in tissues [18]. 

### 4.4. Proximate Composition

The conjugated double bond systems in the benzene rings of phenolic compounds and in the polyene chains of carotenoids are rich in electrons and hydrogen atoms that can be absorbed by the free radicals in the medium, interrupting the sequence of oxidative reactions and ensuring the high antioxidant activity of these bioactive compounds [11,16,85,87,88]. As a result, they reduce the risk of diseases and inflammation [89] and provide better overall health, which reflects in better quality for the final products, as occurred in this study.

Some phenolic compounds act as growth promoters by modulating the animal metabolism in favor of muscle tissue growth and increasing nutrient bioavailability. For example, ascorbic acid and tocopherol, vitamins with antioxidant potential, are preserved and made available for animal metabolism, while phenolic compounds combat free radicals [56]. It is known that oxidative reactions of proteins and lipids lead to decreased digestibility and bioavailability due to changes in their structures [27,28]. So, it is feasible to deduce that as polyphenols and carotenoids prevent the dietary proteins and lipids from oxidation, their structures remain preserved to be used for the animal metabolism, including the production of muscles.

This may explain the composition of the fillets of the tilapias fed the fruit by-products, with higher contents of lipids and protein (fish fed GRM). Fish lipids are known for their high quality, especially due to their content in polyunsaturated fatty acids (PUFA), mainly eicosapentaenoic acid (EPA C20:5 n3), docosahexaenoic acid (DHA C22:6 n3) and linoleic acid (C18:2 n6). The n3 PUFAs, mainly EPA and DHA, are recognized as promoting several benefits to human health, mainly with regard to the prevention of cardiovascular diseases [90].

The control of the oxidative stress promoted by dietary phenolic compounds and carotenoids can also be identified by the lower amount of carbohydrates found in the fillets of fish fed with the fruit by-products, denoting less need for glycogen accumulation as an energy resource to act in stressful situations [91,92]. The moisture and mineral salt contents agreed with data found in the literature, which range from 64% to 90% [93,94] and 1% to 2% [95], respectively.

### 4.5. Lipid Peroxidation

The ice crystals formed during freezing cause tissue and cell ruptures, facilitating the contact between polyunsaturated lipids and the agents of lipid peroxidation and ending with the production of malonaldehyde, one of the main TBARSs formed in this reaction [96]. The low TBARSs found in the fillets of the groups fed the fruit meals proved the antioxidant activity of the phenolic compounds and carotenoids, which was quite evident with 60 days of storage. The best results provided by the apple meal are possibly due to the presence of chlorogenic acid, a phenolic compound with excellent antioxidant capacity [97].

The lipid stability provided by the bioactive compounds has great practical importance, since tilapia meat is one of the most consumed fish worldwide due to its flavor characteristics, appearance and easy preparation [4], and the extension of its shelf life is of interest to all consumers. Moreover, as the carotenoids are deposited in the muscles, the possibility of their transportation to the human diet should not be underestimated, since, in the body, they may exert the same antioxidant actions, bringing benefits to health [15].

The results found in this study indicate good perspectives on the use of fruit by-products in tilapias farming. Perhaps the results could be more evident if the feeding time or the level of inclusion of fruit meals were greater, mainly in terms of growth performance. On the other hand, perhaps higher levels of inclusion could harm these parameters if the by-products contained many antinutrients, which deserves investigation too. In any case, these preliminary findings encourage studies with higher levels of inclusion of fruit by-products in tilapias feed that also look at feeding costs, since, in addition to these benefits, their use can also bring economic advantages to production.

## 5. Conclusions

Acerola, apple and grape by-products had high concentrations of phenolic compounds and carotenoids and high antioxidant activity in vitro. Their inclusion in tilapias’ diets did not affect the productive parameters, maintained or improved the fish health and oxidative status and improved the composition and the lipid stability of the fillets. These results lead us to recommend the inclusion of fruit by-products in farmed tilapia diets due to their proven beneficial effects on animal health and animal product quality. 

## Figures and Tables

**Table 1:** Composition of the experimental diets.

Ingredients	ACM	APM	GRM	C
Corn meal	39.00%	39.00%	39.00%	44.10%
Soybean meal	42.00%	42.00%	42.00%	40.00%
Meat and bone meal	5.58%	5.58%	5.58%	6.99%
Viscera meal	3.00%	3.00%	3.00%	4.00%
Brown rice meal	0.00%	0.00%	0.00%	0.02%
Soybean oil	3.60%	3.60%	3.60%	3.00%
Lysine	0.50%	0.50%	0.50%	0.50%
Methionine	0.10%	0.10%	0.10%	0.10%
Threonine	0.10%	0.10%	0.10%	0.10%
Tryptophan	0.10%	0.10%	0.10%	0.01%
Mineral and vitamin premix *	1.00%	1.00%	1.00%	1.00%
NaCl	0.00%	0.00%	0.00%	0.16%
Vitamin C	0.02%	0.02%	0.02%	0.02%
Acerola meal	5.00%	0.00%	0.00%	0.00%
Apple meal	0.00%	5.00%	0.00%	0.00%
Grape meal	0.00%	0.00%	5.00%	0.00%
Nutrients/energy—Calculated values				
Digestible energy	3100.88	3100.57	3100.01	3100.65
Crude protein (%)	27.309	27.109	27.167	27.379
Digestible energy (%)	24.518	24.769	24.728	25.069
Arginine (%)	21.588	21.588	21.588	2.28
Lysine (%)	22.015	22.015	22.015	2.27
Methionine (%)	0.4908	0.4908	0.4908	0.52
Threonine (%)	12.112	12.112	12.112	1.28
Tryptophan (%)	0.4957	0.4957	0.4957	0.50
Mineral matter (%)	58.689	58.689	58.689	6.63
Calcium (%)	10.472	10.472	10.472	1.26
Phosphorus (%)	0.8365	0.8365	0.8365	0.94
Vitamin C (mg/kg)	608.4	608.4	608.4	608.4

ACM = acerola meal diet; APM = apple meal diet; GRM = grape meal diet; C = control diet. * Composition per kg: iron 20,000 mg/kg; copper 3500 mg/kg; zinc 24,000 mg/kg; manganese 10,000 mg/kg; Iodine 160 mg/kg; selenium 100 mg/kg; cobalt 80 mg/kg; inositol 25 g/kg; vitamin A 2,400,000 IU/kg; vitamin D3 600,000 IU/kg; vitamin E 30,000 IU/kg; vitamin C 60 g/kg; vitamin B1 4000 mg/kg; vitamin B2 4000 mg/kg; niacin 20 g/kg; vitamin B6 3500 mg/kg; vitamin B12 8000 mg/kg; folic acid 1200 mg/kg; biotin 200 mg/kg; pantothenic acid 10 mg/kg; vitamin K3 3000 mg/kg; choline 100 g/kg.

**Table 2:** Bioactive compounds and antioxidant activity of the fruit meals added to the experimental diets (means ± standard deviation).

Bioactive Compounds	Meals			*p*-Value
	ACM	APM	GRM	
TPC (μg/100 mg)	772 ± 7.2 ^b^	261 ± 78.4 ^c^	857 ± 2.6 ^a^	<0.001
Carotenoids (mg/kg)	2.87 ± 0.55 ^b^	4.03 ± 0.56 ^ab^	5.76 ± 0.58 ^a^	0.006
Antioxidant capacity				
ABTS (μM Trolox/g)	20.7 ± 1.55 ^b^	3.4 ± 1.31 ^c^	132.9 ± 4.98 ^a^	<0.001
DPPH (EC50 mg/L)	670 ± 4.03 ^b^	733 ± 5.12 ^a^	463 ± 4.03 ^b^	<0.001

ACM = acerola meal; APM = apple meal; GRM = grape meal; TPC = total phenolic compounds. ABTS = 2,2-azinobis (3-ethylbenzothiazoline-6-sulfonic acid); DPPH = 2,2-diphenyl-1-picrylhydrazyl. Values followed by different letters differ significantly by Tukey’s test (*p* < 0.05).

**Table 3:** Performance parameters of the tilapias fed the experimental diets (means ± standard deviation).

Parameters	Diets				*p* Value
	ACM	APM	GRM	C	
Weight gain (g)	591.00 ± 38 ^a^	491.00 ± 21.9 ^bc^	471.00 ± 32.8 ^c^	531.00 ± 48.8 ^b^	0.002
Feed efficiency	0.095 ± 0.028	0.061 ± 0.039	0.107 ± 0.046	0.095 ± 0.055	0.310
Body condition factor	2.706 ± 0.155	2.666 ± 0.291	2.768 ± 0.172	2.370 ± 0.616	0.198
Hepatosomatic index	1.156 ± 0.344	1.181 ± 0.369	1.275 ± 0.424	1.179 ± 0.403	0.880

ACM = diet with 5% acerola meal; APM = diet with 5% apple meal; GRM = diet with 5% grape meal; C = control diet. Values followed by different letters differed significantly by Tukey’s test (*p* < 0.05).

**Table 4:** Biochemical parameters of the blood of tilapias fed with the experimental diets (means ± standard deviation).

Parameters	Diets				Reference Limits	*p* Value
	ACM	APM	GRM	C		
Total protein (g/dL)	3.59 ± 0.53	3.31 ± 1.16	3.64 ± 0.62	3.71 ± 0.71	3.3–5.0 g/dL	0.538
Albumin (g/dL)	1.31 ± 0.31	1.16 ± 0.47	1.31 ± 0.33	1.46 ± 0.39	1.1–1.7 g/dL	0.166
Globulin (g/dL)	2.27 ± 0.32 ^b^	1.82 ± 0.54 ^a^	2.24 ± 0.30 ^b^	2.25 ± 0.41 ^b^	1.3–3.1 g/dL	0.012
Albumin/globulin	0.58 ± 0.13	0.61 ± 0.18	0.55 ± 0.10	0.66 ± 0.13	0.4–0.8	0.084
Alanine aminotransferase (ALT) (U/L)	81.2 ± 22.4	81.9 ± 13.8	75.2 ± 12.9	84.1 ± 19.2	28.3–121 U/L	0.253
Aspartate aminotransferase (AST) (U/L)	74.7 ± 25.3	75.7 ± 19.4	67.0 ± 12.9	81.7 ± 22.0	16–120 U/L	0.052
Alkaline phosphatase (U/L)	41.9 ± 14.3 ^a^	53.1 ± 19.1 ^ab^	39.5 ± 11.9 ^a^	59.2 ± 28.0 ^b^	16–38 U/L	0.005
Glucose (mg/dL)	59.6 ± 19.0 ^a^	68.0 ± 17.7 ^ab^	87.3 ± 35.0 ^b^	65.1 ± 25.4 ^a^	52–156 mg/dL	<0.001
Cholesterol (mg/dL)	138 ± 23.9	137 ± 45.9	155 ± 40.0	152 ± 31.1	88–228 mg/dL	0.206
Triglycerides (mg/dL)	143 ± 34.0	123 ± 81.9	131 ± 61.7	158 ± 48.7	*	0.376
Uric acid (mg/dL)	2.30 ± 1.04 ^a^	3.23 ± 1.49 ^ab^	2.25 ± 0.88 ^a^	4.04 ± 1.85 ^b^	*	<0.001
Creatinine (mg/dL)	0.154 ± 0.05 ^a^	0.221 ± 0.11 ^b^	0.181 ± 0.06 ^ab^	0.179 ± 0.04 ^ab^	0–0.8 mg/dL	0.031
Urea (mg/dL)	2.73 ± 0.58 ^b^	2.29 ± 0.88 ^ab^	3.55 ± 1.09 ^c^	1.85 ± 0.52 ^a^	1.0–4.0 mg/dL	<0.001
TBARS (ng MDA/mL)	3.96 ± 2.31 ^a^	5.14 ± 3.86 ^a^	3.14 ± 1.83 ^a^	8.51 ± 5.12 ^b^	*	<0.001
TAS (mmol/L)	0.576 ± 0.10 ^a^	0.558 ± 0.06 ^a^	0.594 ± 0.06 ^a^	0.464 ± 0.04 ^b^	*	<0.001
TOS (mmol/L)	0.05 ± 0.006 ^a^	0.05 ± 0.002 ^a^	0.05 ± 0.004 ^a^	0.06 ± 0.003 ^b^	*	<0.001

ACM = diet with 5% acerola meal; APM = diet with 5% apple meal; GRM = diet with 5% grape meal; C = control diet; TBARSs = 2-thiobarbituric acid reactive substances; MDA = malonaldehyde; TAS = total antioxidant status; TOS = total oxidant status. Values followed by different letters differed significantly by Tukey’s test (*p* < 0.05). * No reference values were found.

**Table 5:** Proximate composition of the fillets of tilapias fed the experimental diets (means ± standard deviation).

Parameters	Diets				*p* Value
	ACM	APM	GRM	C	
Moisture (%)	78.18 ± 0.93 ^bc^	79.12 ± 1.21 ^a^	78.78 ± 0.80 ^ab^	77.90 ± 0.94 ^c^	<0.001
Proteins (%)	15.67 ± 0.99 ^ab^	14.85 ± 0.63 ^b^	16.55 ± 0.31 ^a^	15.01 ± 1.4 ^b^	<0.001
Carbohydrates (%)	1.76 ± 0,82 ^b^	1.73 ± 0.80 ^b^	0.57 ± 0.28 ^c^	3.66 ± 1.24 ^a^	<0.001
Lipids (%)	3.17 ± 0.86 ^a^	3.14 ± 0.56 ^a^	2.83 ± 0.12 ^a^	2.21 ± 0.38 ^b^	<0.001
Ashes (%)	1.23 ± 0.74 ^ab^	1.17 ± 0.9 ^b^	1.26 ± 0.06 ^a^	1.28 ± 0.14 ^a^	0.003

ACM = diet with 5% acerola meal; APM = diet with 5% apple meal; GRM = diet with 5% grape meal; C = control diet; values followed by different letters differ significantly by Tukey’s test (*p* < 0.05).

## Data Availability

Not applicable.
